# Gel-free multiplexed reduced representation bisulfite sequencing for large-scale DNA methylation profiling

**DOI:** 10.1186/gb-2012-13-10-r92

**Published:** 2012-10-03

**Authors:** Patrick Boyle, Kendell Clement, Hongcang Gu, Zachary D Smith, Michael Ziller, Jennifer L Fostel, Laurie Holmes, Jim Meldrim, Fontina Kelley, Andreas Gnirke, Alexander Meissner

**Affiliations:** 1 Broad Institute of MIT and Harvard, Cambridge, MA 02142, USA; 2 Harvard Stem Cell Institute, Cambridge, MA 02138, USA; 3 Department of Stem Cell and Regenerative Biology, Harvard University, Cambridge, MA 02138, USA; 4 Harvard-MIT Division of Health Sciences and Technology, Cambridge, MA 02139, USA

## Abstract

Sequencing-based approaches have led to new insights about DNA methylation. While many different techniques for genome-scale mapping of DNA methylation have been employed, throughput has been a key limitation for most. To further facilitate the mapping of DNA methylation, we describe a protocol for gel-free multiplexed reduced representation bisulfite sequencing (mRRBS) that reduces the workload dramatically and enables processing of 96 or more samples per week. mRRBS achieves similar CpG coverage to the original RRBS protocol, while the higher throughput and lower cost make it better suited for large-scale DNA methylation mapping studies, including cohorts of cancer samples.

## Background

DNA methylation plays an important role in mammalian development [1,2] and is frequently altered in diseases, including cancer [3]. It is generally thought that methylation acts in a repressive function within regulatory contexts [4,5]. DNA methylation in mammalian genomes occurs mostly within the context of the CpG dinucleotide [6] and is generally seen in CpG-poor regions. In contrast, CpG-rich regions naturally exhibit low methylation states [7-10].

Many techniques have been developed to investigate global DNA methylation patterns [11]. Comparison of next-generation sequencing-based technologies showed that most methods produce similar results [12,13], but that the optimal sequencing strategy may depend on sample DNA amount, as well as the desired genome coverage and sequencing depth [14,15]. Whole-genome bisulfite sequencing of randomly sheared genomic DNA is the most comprehensive, but also most costly, method, while more focused approaches such as reduced representation bisulfite sequencing (RRBS) allow larger numbers of samples to be analyzed at reduced costs [8,15-17].

RRBS utilizes the cutting pattern of MspI (C^CGG) to systematically digest DNA to enrich for CpG dinucleotides. As opposed to whole-genome bisulfite sequencing, every fragment produced by MspI digestion will contain DNA methylation information for at least one CpG dinucleotide [6]. Another benefit of RRBS is that promoters, CpG islands, and other genomic features are disproportionally enriched genomic features because of the frequency of MspI cut sites in these regions [8,16].

RRBS reduces the complexity of the genome - and thus the sequencing cost - by selecting a subset of MspI fragments based on their size for sequencing. In the standard RRBS protocol, this size selection is done by preparative gel electrophoresis, which is laborious and difficult to automate, thereby limiting the throughput of the method. For example, using our more recently published protocol [15], which includes a manual 40 to 220 bp size cut on an agarose gel, it is possible to produce around 12 to 24 RRBS libraries within a two-week time period. We reasoned that removing MspI fragments <40 bp by a simple clean-up protocol followed by bisulfite conversion, PCR and cluster amplification on an Illumina flowcell (all of which select against large fragments) could result in a similar size distribution of MspI fragments and comparable reduced representation of the genome as in the traditional, gel-based protocol. Taking advantage of increased sequencing throughput and the ability to barcode sequencing libraries, we have developed a new 'gel-free' multiplexed RRBS protocol, called mRRBS, which allows processing of samples in batches of 96 or more.

In addition to multiplexing and skipping the preparative gel, the mRRBS protocol was simplified and streamlined, eliminating several other steps of the original RRBS protocol. For example, the addition of Klenow fragment (3'→5' exo-) directly to the post-digested MspI/DNA mixture for end repair, and adding the A-tail minimizes clean-up steps and loss of material. The replacement of multiple phenol:chloroform steps described in the original RRBS method [8,15] with a single solid phase reversible immobilization (SPRI) bead clean-up after adapter ligation also helped improve the ease and efficiency of the library generation process.

Rapid library generation using mRRBS will greatly increase the throughput while notably reducing the cost per sample. As a proof of concept, we show the generation of 96 libraries using the new mRRBS protocol and provide statistics as well as comparative performance measures of this improved method. To facilitate future large-scale studies we also provide detailed reagent lists and the costs (labor and reagents) per sample.

## Results and discussion

### Streamlining the RRBS protocol

**Figure 1:**
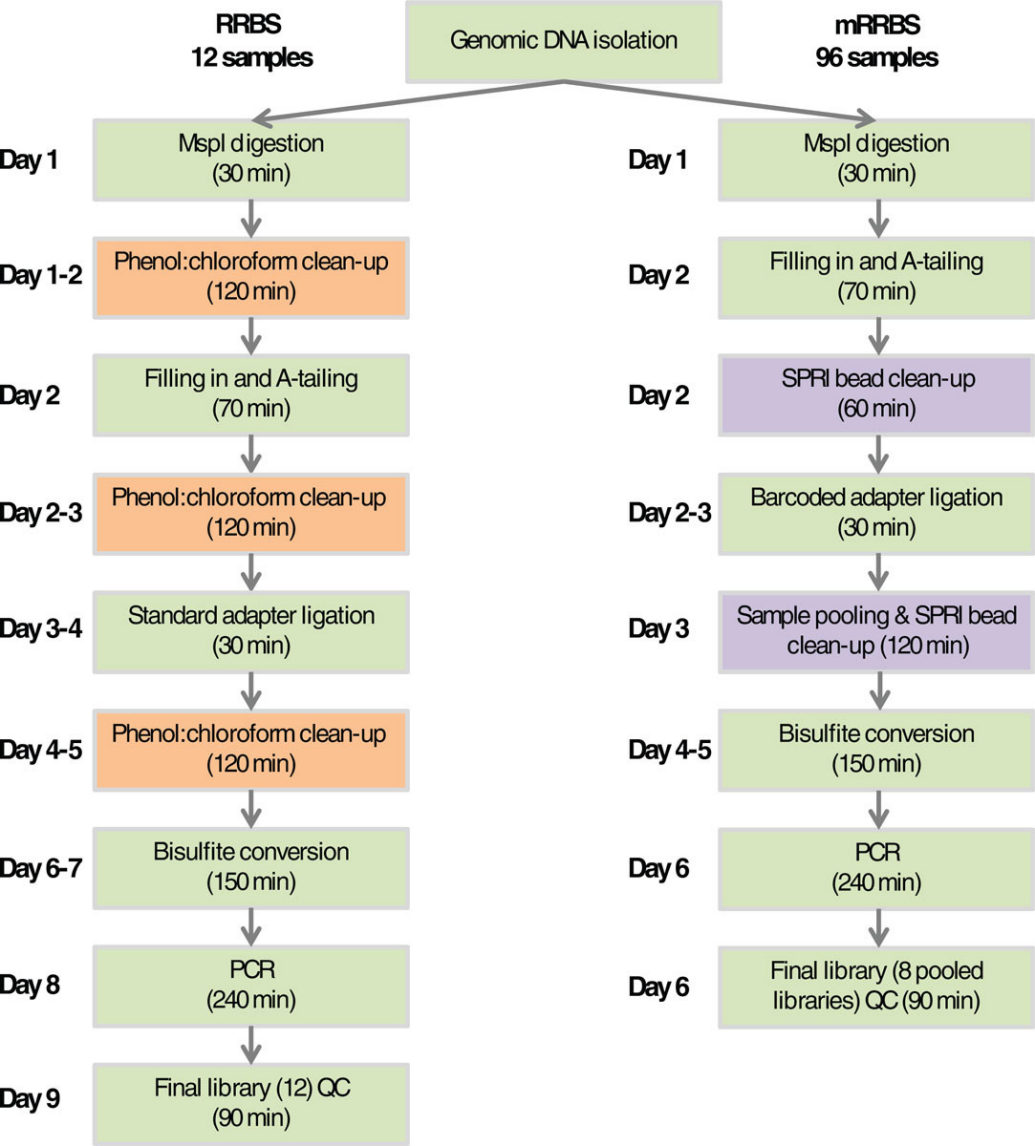
**Flowchart comparing RRBS and mRRBS steps**. Each step that can be completed in a standard workday is shown. Orange boxes highlight phenol:chloroform clean-up and preparative agarose gel purification steps that were omitted in the new mRRBS protocol. Purple boxes highlight key new steps specific to mRRBS. Each box also shows the approximate amount of hands-on time required per step. QC, quality control.

Dramatically reduced next-generation sequencing costs have paved the way for large-scale sequencing projects; however, generating libraries has become the bottleneck in DNA methylation profiling studies. Traditional library preparation is performed using microfuge tubes, which prevents the processing of libraries *en masse*. In addition, gel size-selection of DNA libraries remains a rate-limiting step in RRBS that was designed to produce comparable genome coverage across many samples. In addition to being time-consuming, it is a potential source of sample cross-contamination. In our original protocol [8,15], we reported that one person can make 12 RRBS libraries in 9 days (Figure 1, left) [15]. To simplify this RRBS protocol and to enable its use for large-scale DNA methylation profiling, we modified the protocol as follows (Figure 1, right).

First, enzymatic reactions were processed in a 96-well PCR plate using a 12-channel pipette (Figure S1 in Additional file 1). All 96 samples were quantified, and DNA samples were diluted to an equal concentration (20 ng/μl). DNA (5 μl, 100 ng) from each sample was used for the proof-of-concept experiment. Second, because both MspI digestion and end repair/A-tailing by Klenow fragment (3'→5' exo-) can be carried out in the same buffer, the MspI inactivation and removal steps were eliminated. The DNA purification after A-tailing and adapter ligation used a modified '1 tube with bead' SPRI clean-up method [18] in which several reaction steps are carried out in a single tube. Third, Illumina TruSeq adapters with unique six-base identifiers were used to tag and pool 12 samples early in the process and sequence them later in a single lane of Illumina Hi-Seq. In theory, many more samples can be tagged and processed as a pool. However, at this time only 24 different methylated TruSeq adapters are commercially available. Fourth, we simplified and streamlined the clean-up process. Traditional phenol extraction followed by ethanol precipitation is tedious and time consuming, but DNA purification before adapter ligation using regular spin columns significantly reduces recovery rates of small (<70 bp) DNA fragments. We eliminated these and also skipped the preparative electrophoresis step, relying on SPRI bead clean-up to remove small MspI fragments and bisulfite-induced DNA fragmentation, and amplification bias to select against unwanted large fragments. Together, these modifications reduce library processing time by about two days (Figure 1; Figure S1 in Additional file 1).

To reduce the occurrence of adapter dimers, we used a lower concentration of adapters (30 nM) than recommended by the manufacturer (see Materials and methods for details). In addition, we extracted the library DNA after the final PCR using two subsequent rounds of SPRI bead clean-ups to minimize primer-dimers in the final libraries (Figure S2 in Additional file 1).

Finally, to overcome density limitations and problems with cluster localization on the Illumina Hi-Seq flowcell due to non-random distribution of bases at the beginning of each read (each read starts with a C or a T, depending on the methylation status of the MspI site, followed by two Gs in a row), we implemented a custom Illumina sequencing protocol called 'dark sequencing'. In this custom protocol, no image is recorded during the first three sequencing cycles and cluster localization is deferred to cycles 4 through 7 (Figure S3 in Additional file 1). After cluster definition, the sequencing primer with seven newly synthesized bases attached is melted off and washed away. Fresh sequencing primer is annealed and the crucial first position that indicates the methylation status of the MspI site is determined at the beginning of a new 29-base read (see Materials and methods for details).

### Validation of gel-free mRRBS libraries

We constructed 96 mRRBS libraries from one plate of DNA samples. When assayed on a gel, the size distribution of the final pooled PCR-amplified libraries was similar to that of the original RRBS protocol [8,15] (Figure S2 in Additional file 1).

**Figure 2:**
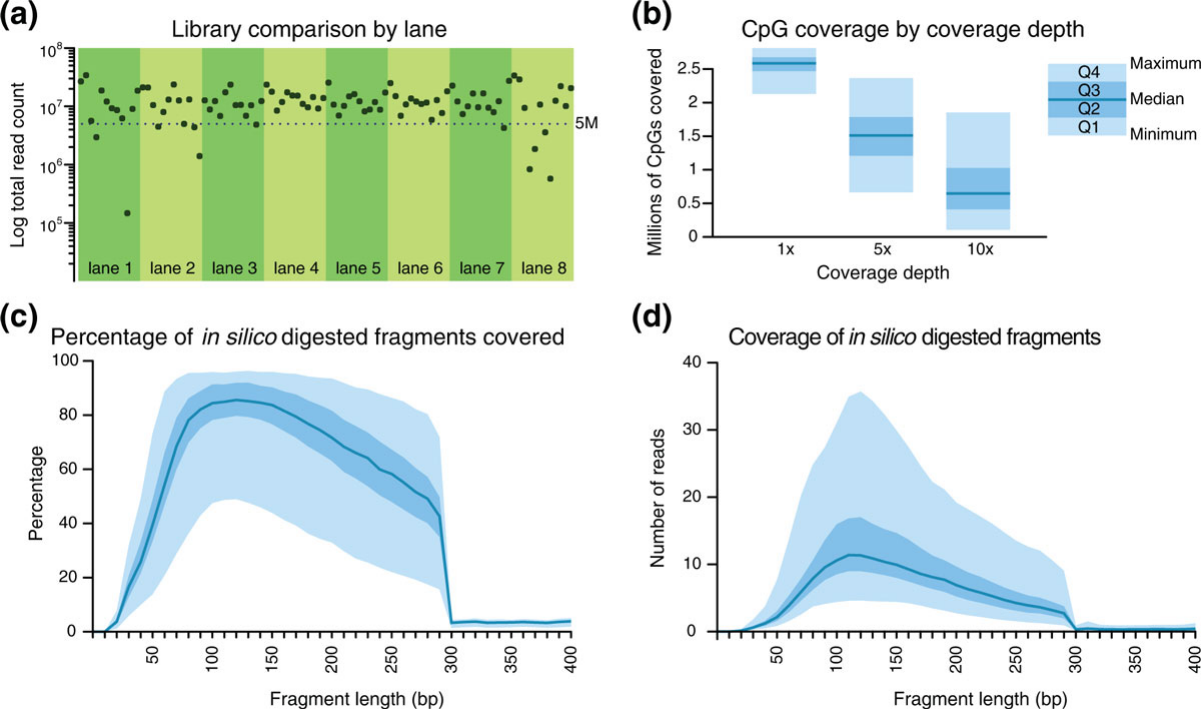
**Performance summary of mRRBS**. Ninety-six samples were processed using mRRBS and sequenced with eight lanes of Illumina HiSeq 2000 using 12 barcoded adapters per lane. **(a) **The total number of reads for each sample is shown 84 samples with >5 million total reads were included in the subsequent comparisons. **(b) **Quartile plots of summary coverage depth from these samples. The minimum and maximum values are bounded by the light blue area in (b-d), while the darker blue area represents the interquartile range. The dark blue line indicates the median. **(c,d) **MspI *in silico *digestion of the hg19 genome produced a total of 1,124,739 fragments. (c) The percentage of fragments of each fragment size that were covered by at least one read. (d) The average coverage depth for fragments of each length. Genomic MspI-digested fragments longer than 300 bp were not included in the sequence alignment target, which partly contributes to the sharp drop in coverage at 300 bp in (c,d).

To evaluate the performance of the mRRBS protocol, we sequenced the 96 libraries using 8 lanes of an Illumina HiSeq 2000 sequencer with 12 libraries per lane, which produced a median of 11.3 million reads per library (Table 1 and Figure 2a; Additional file 2). We used a cutoff of libraries with at least 5 million reads to select 84 high-quality samples with a median read count of 12.2 million, of which a median of 8.92 million passed aligner quality controls, mapped uniquely to the genome and contained information for at least one CpG. We calculated the number of distinct CpGs covered at different depths (1×, 5× and 10×) in these 84 passing samples. As shown in Figure 2b, the majority of samples had >1 million distinct CpGs covered at 5× and >0.5 million CpGs covered with 10 or more reads (Figure 2b). More than 2 million unique CpGs were covered by at least one read. This is comparable to the CpG coverage in the original RRBS protocol [8,15], which had a median 1× coverage of 1.9 million (Table 2).

**Table 1:** Summary of mRRBS performance

Description	Total reads	Informative reads	Bisulfite conversion	1× coverage CpG count	5× coverage CpG count	10× coverage CpG count
96 samples	11,295,879	8,921,543	99%	2,523,793	1,399,192	563,980
84 HQ samples	12,151,833	9,629,839	99%	2,583,636	1,510,414	645,828

The first row summarizes statistics for all 96 libraries generated using mRRBS, and the second row includes only those high-quality (HQ) samples with greater than 5 million reads per sample (see Additional file 2 for per-sample details). The total reads column gives the median number of sequencing reads produced for each library. The number of those reads that passed sequencer quality controls, were aligned to the reference genome, and included in the informative read count (median value). The estimated bisulfite conversion rate is based on all methylated cytosines in a non-CpG context [6]. The median numbers of CpGs covered with at least 1×, 5×, and 10× coverage is shown.

**Table 2:** Summary for 12 RRBS and 12 mRRBS libraries

Description	Total reads	Informative reads	Bisulfite conversion	1× coverage CpG count	5× coverage CpG count	10× coverage CpG count
12 RRBS samples	18,066,460	12,482,608	99%	1,851,441	1,312,909	831,581
12 mRRBS samples	12,523,362	10,000,051	99%	2,631,436	1,617,861	704,994

**Figure 3:**
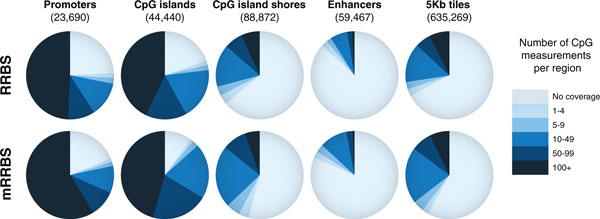
**Comparison of CpG measurements in RRBS (top) and mRRBS (bottom) across five genomic features**. Pie charts compare the relative CpG coverage for different genomic features as sampled by the original RRBS and mRRBS protocol. Twelve representative samples with 10 to 20 million reads and more than 10 million mapped reads were selected from each method (Table 2; Additional file 2). The number of unique CpG measurements residing within a given feature must be observed in at least 80% of the samples used to be scored at a given coverage. Promoters are defined as 1 kb upstream and 1 kb downstream of the transcription start site of Ensembl genes. CgiHunter was used to computationally derive CpG islands with a minimum CpG observed versus expected ratio of 0.6, a minimum GC content of 0.5 and a minimum length of 700 bp. CpG island shores are defined as the 2 kb regions adjacent to the derived CpG islands. Previously published H3K4me2 peaks across multiple human cells were used to derive a consensus enhancer set [20]. As a more global measurement, the genome was divided into non-overlapping consecutive 5 kb tiles, and the number of CpG measurements in each tile was analyzed.

The same statistics reported in Table 1 are shown here for 12 RRBS and 12 mRRBS samples that were used for the coverage comparison in Figure 3.

Because the mRRBS protocol avoids the gel size selection step, *in silico *analysis was used to determine coverage rates for different sizes of fragments. The hg19 human genome was digested with MspI *in silico*, and the resulting fragments were binned by size. In Figure 2c, we measured the percentage of fragments of each size that were covered by at least one read. Fragments with a size range of 60 to 300 bp appear well-covered in most samples, with a slight, PCR-induced bias toward fragments of shorter length. In Figure 2d, coverage depth is shown for corresponding fragment sizes. For each bin of fragments with a certain length, the average coverage of all fragments of that size is reported, though this likely underestimates the actual average CpG coverage because artificially digested fragments that are not covered by any sequencing reads are included in the mean coverage calculation. On average, fragments with a size range of 60 to 300 bp are covered at least 5-fold, which is the recommended coverage threshold [8,12]. Indeed, CpGs captured with at least 5× coverage correlate highly between sequencing runs of the same sample, whereas correlation between CpGs captured with a lower coverage show a lower correlation (Figure S4 in Additional file 1).

### Comparison of genomic coverage

We next selected 12 previously generated RRBS libraries for comparison with 12 mRRBS libraries (Table 2; Additional file 2). In order to increase comparability, we chose only samples with 10 to 20 million total reads and greater than 10 million aligned reads. To reduce the biases of size selection, we also selected original RRBS libraries with a wide size selection of between 30 and 280 bp. We next counted the number of individual CpG measurements for five distinct features: (1) promoters, (2) CpG islands, (3) CpG island shores, (4) enhancers and (5) whole-genome 5 kb tiles (summarized in Figure 3). The comparison highlights that coverage is comparable between mRRBS and the original RRBS protocol [8,15]. The mild increase in coverage for some features in mRRBS may be a consequence of the broader size range that allows for more sequenced fragments at the lower (<30 bp) and higher (>280 bp) end of the spectrum. In addition to these genomic features we determined the coverage of repetitive elements. Approximately 11.6% of mRRBS reads align to repeats, and the vast majority (77%) of repeat hits are SINE/7SL elements. A detailed breakdown of the fraction of reads that align to various classes of repeat elements is shown in Figure S5 in Additional file 1.

**Figure 4:**
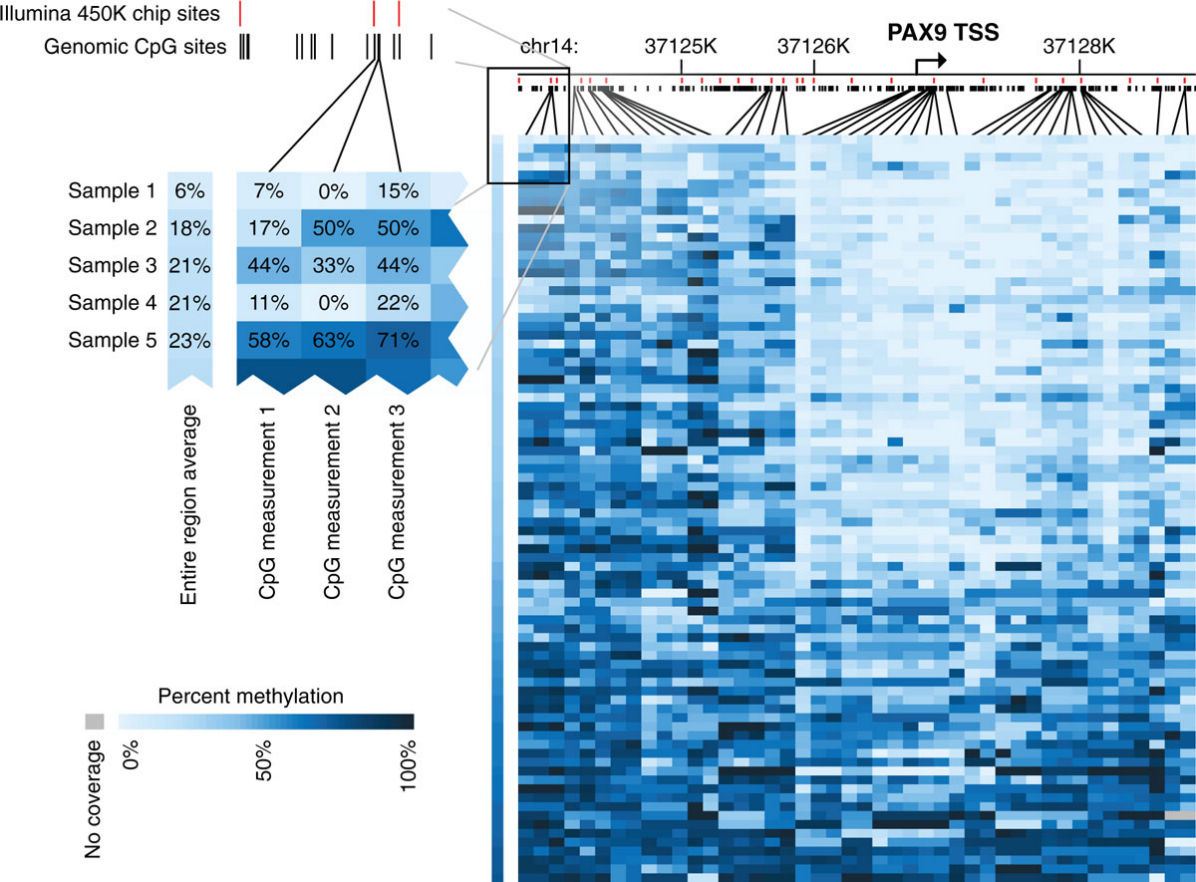
**Single-base resolution view across the *PAX9 *locus**. DNA methylation values of 44 individual CpGs that are captured at greater than 5× coverage within at least 80% of our 84 high-quality samples are shown for the region 3 kb upstream and 2 kb downstream of the *PAX9 *transcription start site. The 279 genomic CpGs within this region are marked in black and those captured by the Illumina Infinium HumanMethylation450 BeadChip Kit are shown in red. The regional average of these 44 CpGs is shown to the left of the individual CpG measurements for each sample.

Figure 4 shows a representative example of the single-base-pair resolution by mRRBS across multiple samples with remarkable cross-sample comparability. The detailed methylation map of the *PAX9 *locus indicates diverse methylation levels for different regions among the 84 analyzed samples, while still covering nearly twice as many CpGs as the Illumina 450K microarray (red bars in Figure 4). These samples were selected for the proof-of-concept experiment due to availability of the DNA, and any biological interpretation of the DNA methylation differences is avoided within this technical report.

### Assessment of PCR-induced chimeras

Barcoding DNA samples early in the process and bisulfite-converting and PCR-amplifying them as a pool contributes significantly to the overall ease and efficiency of the mRRBS protocol. However, pooling prior to PCR carries the risk of cross-sample confusion by chimeric events that cause reads from one sample to be associated with the barcode of another sample.

To assess the magnitude of this potential problem, we prepared a barcoded mRRBS library from *in vitro *CpG-methylated mouse DNA (95% of mRRBS reads were completely methylated) and a barcoded library from wild-type mouse DNA where many regions are far less methylated (45% of mRRBS reads were completely unmethylated). As shown in Figure S6 in Additional file 1, PCR-amplifying both libraries as a pool did not change the proportion of completely methylated, completely unmethylated and partially methylated reads assigned to each sample based on its respective barcode. This indicates a low or undetectable rate of PCR-induced chimerism in mRRBS libraries that does not affect interpretation or analysis of the generated libraries.

Moreover, when calculated, the rate of chimeric artifacts that join unrelated genomic loci in both RRBS and mRRBS data sets is extremely low. In the twelve mRRBS used to compare genomic coverage above, the average rate of one or more mismatches in read alignment was only 1.4 × 10^-5^. This indicates that such disruptive chimeras happen very infrequently.

### Cost reduction and protocol efficiency

Instead of sequencing one sample per lane, which when using newer sequencing platforms such as the HiSeq 2000 produces excessive sequencing reads, mRRBS harnesses barcoded multiplexing technology to reduce sequencing cost and increase efficiency. Table 3 compares the cost of the traditional RRBS method to the new mRRBS method. The costs of all consumables in the lab were added to the total based on their list prices posted on the manufacturers' websites. The cost of adapters purchased from Illumina (see Materials and methods) and the costs of sequencing were added to the price of the lab supplies. The sum of the reagent, sequencing, and estimated salary expenses projected to produce 96 mRRBS libraries in parallel is about half of that described for the original RRBS protocol when examined on a per sample basis [8,15].

**Table 3:** Cost comparison of RRBS and mRRBS

		mRRBS	RRBS
Enzymes	Total (96 samples) enzymes	$665.99	$998.69
	Per sample	$6.94	$10.40
Other supplies and sequencing^a^	Total (96 samples) other supplies and sequencing	$16,770.00	$15,360.00
	Per sample	$174.69	$160.00
With salary^b^	Total (96 samples) supplies + salary	$18,820.60	$37,254.08
	Total per sample cost	$196.05	$388.06

^a^Sequencing costs are based on the current list price for HiSeq 40 bp indexed single read at the Whitehead Institute Core Facility. ^b^Using the previous RRBS method, an estimated 72 days are required to complete library preparation for 96 samples, whereas only 6 days are required using the mRRBS method. Salary costs are calculated using these labor estimates with a $60,000 annual research associate salary. Values are US dollars.

## Conclusions

The mRRBS protocol presented here shortens the time required to produce bisulfite-converted libraries from 9 days in our previous RRBS protocol [15] to around 6 days (Figure 1). Moreover, by performing all initial library assembly steps in 96-well plates and by using unique, per sample, barcoded adapters, many more samples can be processed in parallel, making it possible to generate hundreds of libraries per month. The multiplexing adapters and Illumina Hiseq 2000 technology enable the sequencing of a dozen or more libraries per lane and substantially reduce the per sample cost. Despite these protocol modifications and simplifications, the coverage remains comparable to that described for the original RRBS protocol [8,15], with a below threshold rate (<5 million aligned reads) in our proof of concept plate that is clearly acceptable given the higher throughput and lower cost when generating and sequencing libraries in parallel. DNA quantity or quality may be responsible for some of the observed variability in individual library performance and remains a critical part of the RRBS protocol that likely has stronger effects when using a multiplexed strategy. In summary, mRRBS allows throughputs comparable to array-based platforms such as the Illumina 450k, at a reduced cost, with better genomic coverage and lower genomic DNA input.

## Materials and methods

### Genomic DNA purification

Genomic DNA was isolated as previously reported [15,19]. Purified DNA was quantified using a Quant-iT DNA Broad Range assay kit (Invitrogen, Grand Island, NY, USA, catalogue number Q-33130) and subsequently diluted to 20 ng/μl in low TE (10 mM Tris-HCl, 0.1 mM EDTA, PH 8.0). Equal amounts of DNA samples (100 ng) were added to distinct wells in a 96-well PCR plate (Axygen, Union City, CA, USA, catalogue number PCR-96M2-HS-C). For the chimera experiment, CpG Methylated NIH 3T3 mouse genomic DNA was purchased from New England Biolabs (Ipswich, MA, USA).

### MspI digestion

Samples of 5 μl genomic DNA were transferred to a new 96-well PCR plate with a 12-channel pipette. The MspI (New England Biolabs, catalogue number R0106L) digestion was conducted in a 30 μl reaction containing 3 μl of 10× NEB buffer 2, 1 μl of MspI (20 U/μl) and 21 μl H_2_O. To facilitate pipetting, a master mixture for 110 reactions, which compensates for reagent loss, was set up as follows: 330 μl of 10× NEB buffer 2, 110 μl of MspI and 2,310 μl of H_2_O. Next, 220 μl of the master mixture was added to each of the 12 wells in a row of a 96-well plate. Out of these, 25 μl were then pipetted to the sample/DNA plate using a 12-channel pipette. After carefully sealing the plate with one piece of adhesive tape sheet (Qiagen, Valencia, CA, USA, catalogue number 19570), the plate was then spun down briefly, vortexed to mix and was further spun for 30 s at 2,000 rpm in a PCR plate centrifuge. The plate was then incubated overnight at 37°C in an incubator. A diagnostic gel can be run on select samples at this point to determine MspI digestion efficiency, although this is usually not necessary (Figure S2a in Additional file 1).

### Gap filling and A-tailing

Without deactivating MspI and cleaning-up the digestion reactions, DNA end repair and A-tailing were conducted by adding Klenow fragment (3'→5' exo-) (New England Biolabs, catalogue number M0212L) and dNTP mixture containing 10 mM dATP, 1 mM dCTP and 1 mM dGTP (New England Biolabs, catalogue number N0446S) directly into each well of the digestion plate. To simplify pipetting, an excessive amount of master mixture (110×) containing 110 μl of the Klenow fragment (3'→5' exo-) and 110 μl of the dNTP mix was made, and an aliquot of 18 μl was pipetted to each of the 12 wells in a clean row of a 96-well plate; 2 μl of that mix was added to each sample using a 12-channel pipette. Next, the sample plate was sealed and spun briefly to bring down any liquid accumulated on plate walls. The plate was vortexed to mix and spun for 30 s at room temperature using the plate centrifuge. The reaction was performed in a thermocycler (Eppendorf, Mastercycler EP Gradient S) without the heated lid. The program was set to 30°C for 20 minutes, 37°C for 20 minutes then 4°C indefinitely. The two temperatures are necessary for each step, the gap filling and the A-tailing, to facilitate both reactions.

A 2× concentration of SPRI AMPure XP beads (Beckman Coulter, Brea, CA, USA, catalogue number A63881; 64 μl beads for 32 μl sample) were added to each well using an 8-channel pipette. Beads and samples were mixed by pipetting up and down at least five times. Then, the mixtures were incubated at room temperature for 30 minutes. After DNA binding, the 96-well plate was placed onto a DynaMag™-96 Side magnet (Invitrogen, catalogue number 123-31D) for 5 minutes. The supernatant was carefully removed from the side opposite the accumulated beads, and the beads were then washed twice with 100 μl of 70% ethanol. Five minutes after the second wash, the ethanol was removed, and the duplex of the plate and the DynaMag™-96 Side magnet was put into a fume hood to dry the beads for 10 minutes. After drying of the beads, 20 μl of EB buffer (New England Biolabs, catalogue number B1561) was added to each well using an 8-channel pipette. The plate was then covered with a new tape sheet, vortexed to resuspend DNA, and spun down as described previously.

### Multiplexed adapter ligation

A 110× ligation master mix was made for 96 reactions as follows: 330 μl of 10× T4 ligation buffer, 110 μl of T4 ligase (New England Biolabs, catalogue number M0202M), and 440 μl of H_2_O (1× volume: 3 μl of 10× T4 ligation buffer, 1 μl of T4 Ligase, 4 μl of H_2_O). Master mix (72 μl) was added to each of the 12 wells in a clean row of a 96-well plate. Next, 18 μl of each Illumina TruSeq adapter (Illumina, Dedham, MA, USA, catalogue number PE-940-2001; from a 1:20 diluted 9 μM stock) were added to corresponding wells in the row (Illumina TruSeq adapters contain 5 mC instead of C and can therefore be used for RRBS). After mixing the adapter-ligase mixtures, 10 μl of each was distributed to correlated samples using a 12-channel pipette. This brought the ligation reaction volume of each sample to 30 μl. The plate was placed into a thermocycler and incubated at 16°C overnight without the heated lid- the heated lid could potentially destroy the ligase.

### Library pooling and bisulfite conversion

After ligation the plate was removed from the thermocycler and the beads were resuspended. Next, the plate was placed back into the thermocycler, and the enzyme was deactivated at 65°C for 20 minutes. It is important to note that the beads need to be resuspended prior to enzyme deactivation because resuspension is difficult after heating to 65°C. Samples were then pooled into eight 1.5 ml microfuge tubes. To bind the DNA back to the beads, a 2× solution (720 μl) of 20% polyethylene glycol (8,000 g/mol), 2.5 M NaCl was added to each tube. The samples were mixed and incubated at room temperature for 30 minutes to ensure maximum binding. After incubation, the samples were put onto a DynaMag™-2 magnet (Invitrogen, catalogue number 123-21D) and incubated for 5 minutes to allow bead attraction to the magnet. The liquid was removed, and the beads were washed with 1.0 ml of 70% ethanol. After removing the ethanol, the tubes were placed in the fume hood to dry the beads until cracks were observed (taking about 30 to 50 minutes). For eluting DNA from the beads, 25 μl of EB buffer was added to each tube; the tubes were vortexed for 20 s and were then centrifuged briefly. The tubes were placed back onto the magnet and the eluent (about 23 μl) was transferred to a new 1.5 ml microfuge tube. About 2 μl is lost due to adherence to the beads, and 3 μl of each sample was set aside for the ligation efficiency test by PCR as described previously [15], except that 0.3 μM of TruSeq primers (forward primer, 5'-AATGATACGGCGACCACCGAGAT-3'; reverse primer, 5'-CAAGCAGAAGACGGCATACGA-3'; Integrated DNA Technologies, Coralville, IA, USA) were utilized.

The remaining 20 μl samples were put through two consecutive bisulfite conversions, and bisulfite converted DNA was cleaned up as described in [15]. After determining the optimized PCR cycle number for each sample, a large-scale PCR reaction (200 μl) for each sample was performed as recommended [15].

### Final SPRI bead clean-up

After the PCR was completed, each well was pooled into a 1.5 ml tube. A 1.2× SPRI bead clean-up (240 μl SPRI beads into a 200 μl library pool) as mentioned above was conducted to remove PCR primers and adapter dimers. The DNA was eluted in 40 μl of EB buffer. To minimize adapter dimers, a second round of SPRI bead clean-up was performed at 1.5× (60 μl SPRI beads into a 40 μl library pool). The final library DNA samples were eluted with 40 μl EB buffer. The pooled libraries were quantified using a Qubit fluorometer (Invitrogen catalogue number Q32857) and a Quant-IT dsDNA HS assay kit (Invitrogen catalogue number Q-33120), and the qualities were determined by running a 4 to 20% Criterion precast polyacrylamide TBE gel (Bio-Rad, Waltham, MA, USA, catalogue number 345-0061). An equal quantity of starting genomic DNA prevents a bias toward more concentrated libraries, so accuracy in these measurements is imperative for sequencing success. The samples were sequenced on an Illumina Hiseq 2000 machine at the Broad Institute Sequencing Platform.

### Sequencing

The MspI recognition cut site (C^CGG) creates fragments that will make the first three bases of every read non-random. This would result in high apparent cluster density, poor DNA cluster localization, and significant data loss during sequencing on the Illumina HiSeq 2000. To improve performance of these samples and increase coverage obtained, we used a method referred to as 'dark sequencing' in which imaging and cluster localization were delayed until the fourth cycle of sequencing chemistry, beyond the extent of bias from the MspI cut site (Figure S3 in Additional file 1).

To do this, we loaded a HiSeq 2000 with a custom recipe file co-developed with Illumina plus extra reagents to support primer re-hybridization. The custom recipe created a new initial 'template read' in which the first three biased bases were incorporated without imaging, followed by four cycles that were incorporated, imaged, and used by the sequencer for cluster localization. Next, the recipe removed the newly synthesized strand using NaOH and a buffer wash, re-hybridized fresh sequencing primer to the sample, and began read 1 data collection as usual from the first base but using the pre-existing cluster map or 'template' generated by the template read. HiSeq Control Software (HCS) provided by Illumina prevented cluster intensity files from the template read to enter downstream analysis.

As all custom chemistry steps were defined by the recipe, this workflow required very little additional hands-on time compared to a standard HiSeq run setup. The template read took approximately 6 h and consumed seven cycles of sequencing reagents prior to the start of data collection. Additional reagents to support re-hybridization after the template read were loaded at the beginning of the run alongside other read 1 and index read sequencing reagents. The following positions differed from the standard setup for an indexed single read run: Pos 16, 3 ml Read 1 Sequencing primer; Pos 18, 5 ml 0.1 N NaOH, Pos 19, 6 ml Illumina wash buffer.

### Alignment

After the removal of adapters and barcodes, 29 bp reads were aligned to the hg19 genome using MAQ. CpG methylation calling was performed by observing the bisulfite transformation in the read as opposed to the genome sequence.

### Accession codes

RRBS data have been deposited at the Gene Expression Omnibus (GEO) under accession [GSE40429].

## Abbreviations

bp: base pair; EB: elution buffer; mRRBS: multiplexed RRBS; PCR: polymerase chain reaction; RRBS: reduced representation bisulfite sequencing; SINE: short interspersed repetitive element; SPRI: solid phase reversible immobilization; TBE: Tris/borate/EDTA.

## Competing interests

The authors declare that they have no competing interests.

## Authors' contributions

PB, KC, HG, AG and AM designed the mRRBS protocol and workflow. PB, HG, ZDS performed the experiments. KC and MZ performed analysis. JLF, LH, JM and FK implemented the dark sequencing. PB, KC, HG, AG and AM wrote the paper with assistance from the other authors. All authors have read and approved the manuscript for publication.

## Supplementary Material

Additional file 1 **Figures S1 to S6**. Figure S1: schematic of the mRRBS protocol. Figure S2: gel images from MspI digested DNA and final pooled libraries. Figure S3: schematic of the dark sequencing approach. Figure S4: pairwise correlation of single-CpG methylation data between technical replicates at different read depths. Figure S5: breakdown of repeat elements captured by mRRBS reads. Figure S6: assessment of rate of chimerism during PCR amplification of barcoded RRBS libraries. Click here for file

Additional file 2 **Supplementary Table 1**. Summary of sequencing results, conversion rates and CpG methylation coverage as well as details for the RRBS versus mRRBS comparison. Click here for file
