# Supplementary material for: Gel-free multiplexed reduced representation bisulfite sequencing for large-scale DNA methylation profiling
Source: Genome Biol. 2012 Oct 3;13(10):R92. doi: 10.1186/gb-2012-13-10-r92 (PMC3491420; doi:10.1186/gb-2012-13-10-r92)
Supplement: Additional file 1 — Figures S1 to S6. Figure S1: schematic of the mRRBS protocol. Figure S2: gel images from MspI digested DNA and final pooled libraries. Figure S3: schematic of the dark sequencing approach. Figure S4: pairwise correlation of single-CpG methylation data between technical replicates at different read depths. Figure S5: breakdown of repeat elements captured by mRRBS reads. Figure S6: assessment of rate of chimerism during PCR amplification of barcoded RRBS libraries. [file gb-2012-13-10-r92-S1.pdf]

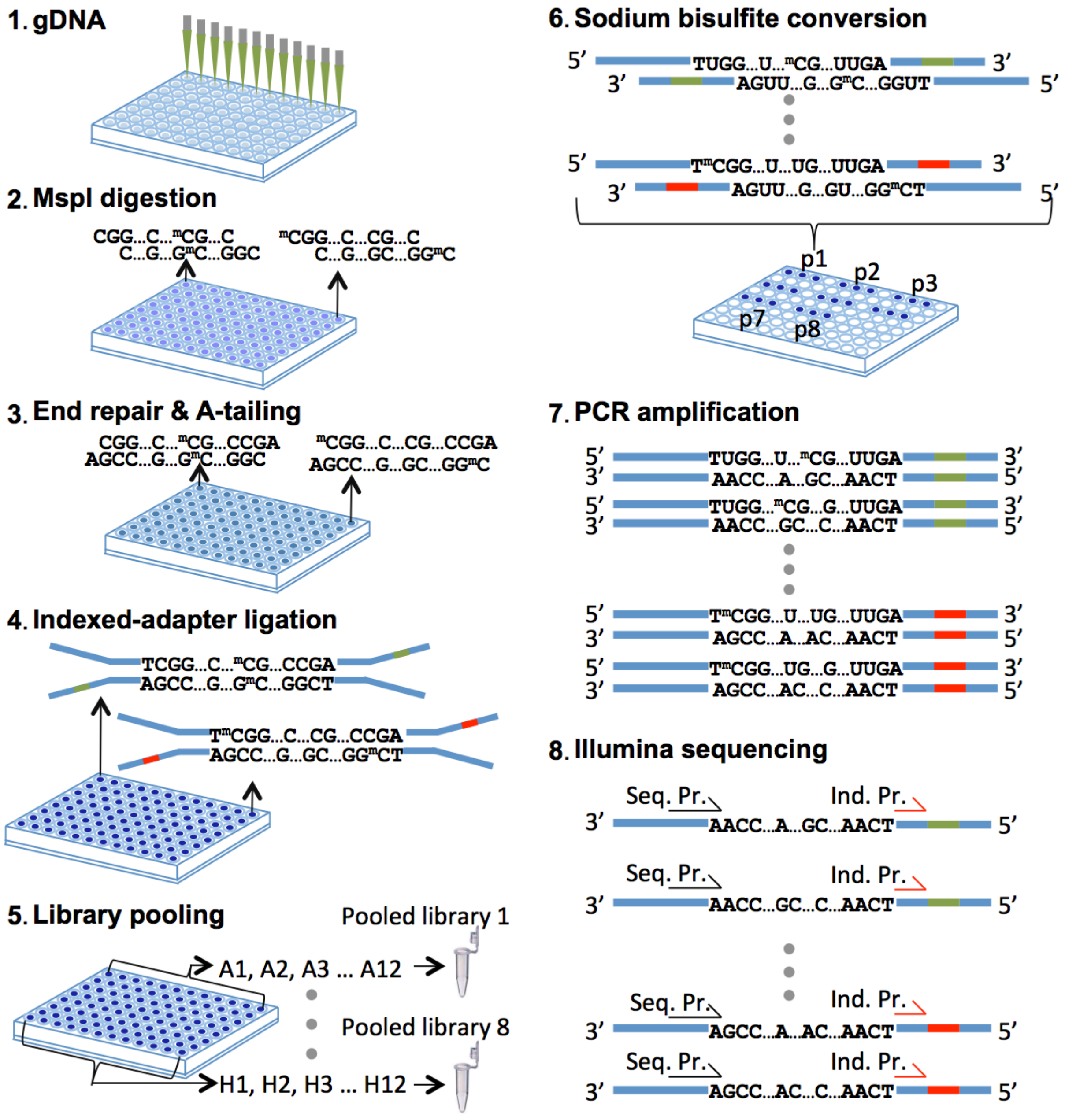

Supplementary Figure 1: Schematic of the mRRBS protocol. Two key steps that were modified and notably reduced the time for library generation were the end repair and adapter ligation. The end repair was carried out in the MspI digestion solution post-digestion, eliminating a phenol:chloroform clean-up step. Adding SPRI beads before the ligation step eliminated the second phenol:chloroform clean-up used in the original protocol (see Figure 1).

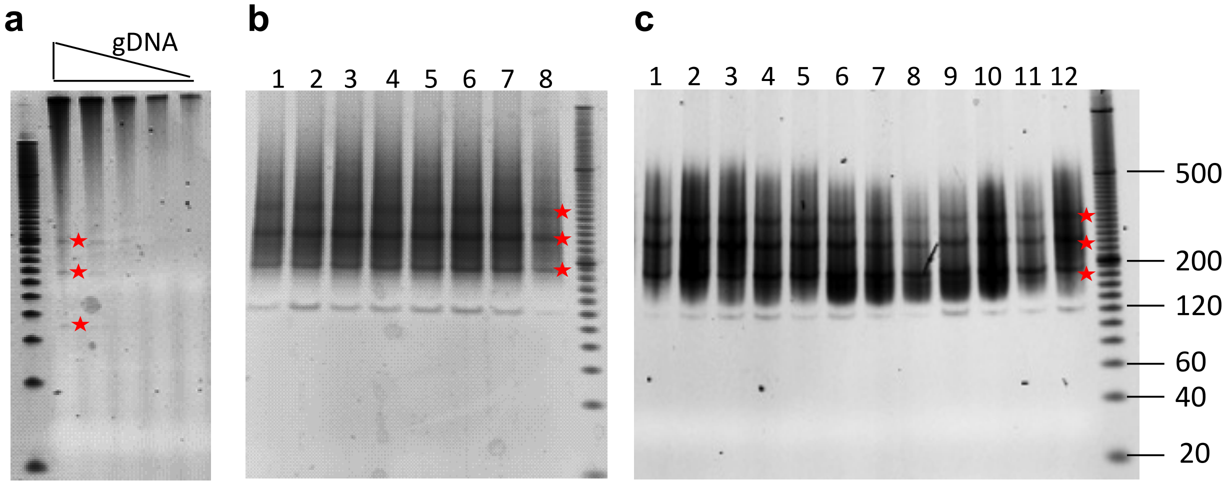

Supplementary Figure 2: Gel images from MspI digested DNA and final pooled libraries. a) MspI digest of human genomic DNA using decreasing quantities of starting material on a 10% TBE PAGE gel. 200 ng, 100 ng, 50 ng, 25 ng and 12.5 ng (left to right) genomic DNA were used for the digest. Note that the faintest smear is barely visible due to low DNA quantity. This amount of DNA will still produce good RRBS libraries using the original protocol [10]. Quantities below 100 ng of genomic DNA have not been tested with the mRRBS protocol. **b**) Gel image of 8 finished pooled mRRBS libraries of 12 samples each (96 samples) run on a 4-20% TBE PAGE gel. **c**) 12 individual RRBS libraries using the original protocol with gel extraction.

Red asterisks mark microsatellite bands typically found in MspI digested DNA. Selected sizes for the DNA ladder are highlighted on the right. All gels used the same DNA ladder.

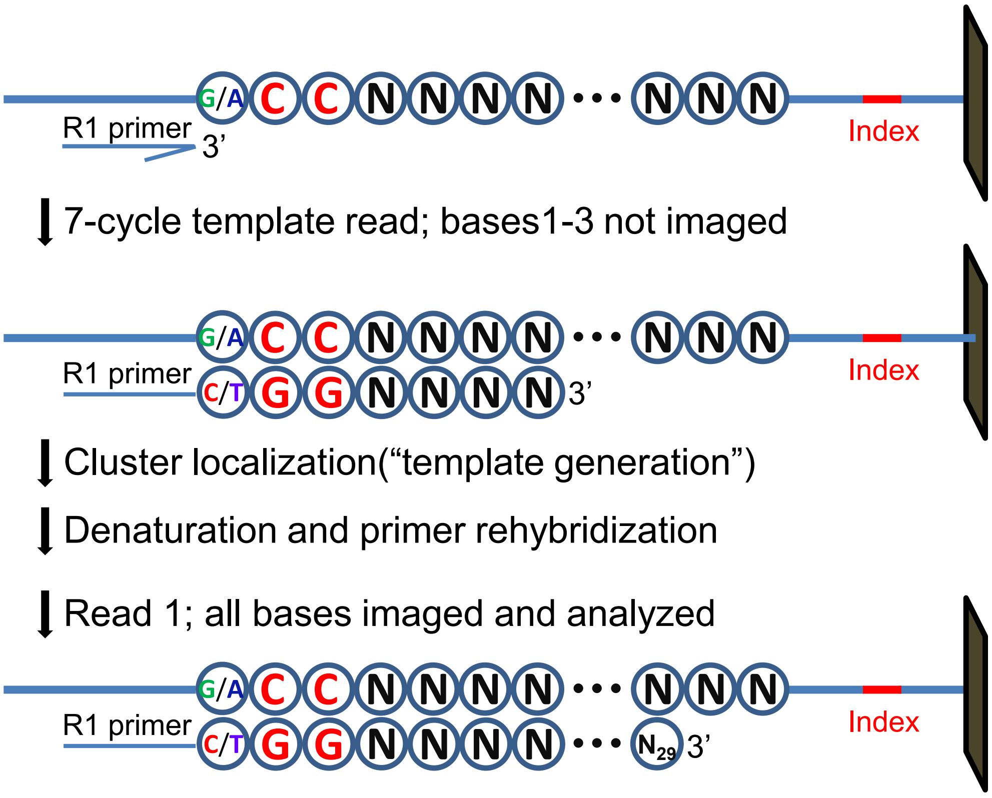

Supplementary Figure 3: Schematic of the dark sequencing approach. The Read 1 (R1) sequencing primer anneals and sequencing chemistry is applied, with the camera turned off during the first 3 cycles. The camera is turned on, and clusters are localized during sequencing cycles 4 through 7. The primer with the 7 newly synthesized bases is melted off and washed away. Fresh sequencing primer is hybridized to the beginning of the strand, and the first position which indicates the methylation status of the MspI site is determined at the beginning of a new 29-base read, using the cluster localization determined during the previously aborted 7-cycle partially "dark" sequencing read.

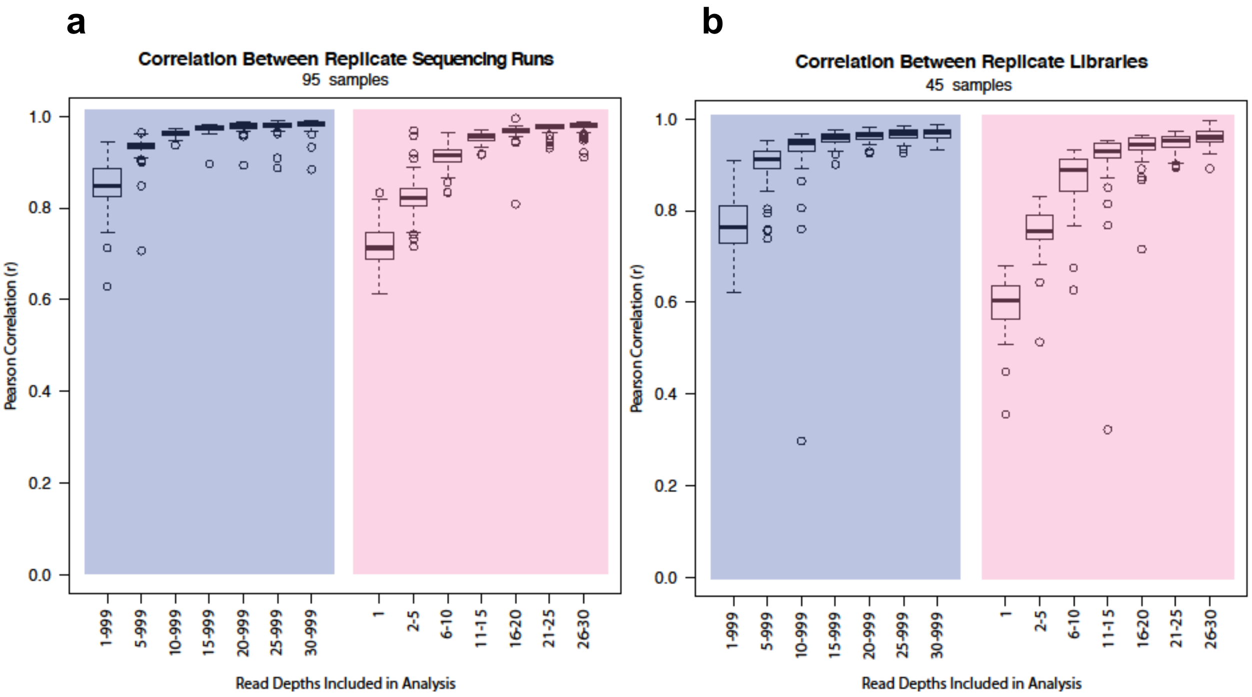

Supplementary Figure 4: Pairwise correlation of single-CpG methylation data between technical replicates at different read depths. Box plots show Pearson correlations of CpG-methylation levels between replicate sequencing runs from a set of 95 mRRBS libraries (**a**) and between replicate library preparations of 45 DNA samples (**b**) for CpGs covered at the read depths shown on the x-axis. The values in the purple-shaded area show the correlation between replicates when different minimum cutoffs were used. The values in the pink-shaded area show the correlation when only reads with a certain coverage are used. For example, the left-most box plot in the pink-shaded area shows the correlation between replicates when only CpGs with 1x coverage are used.

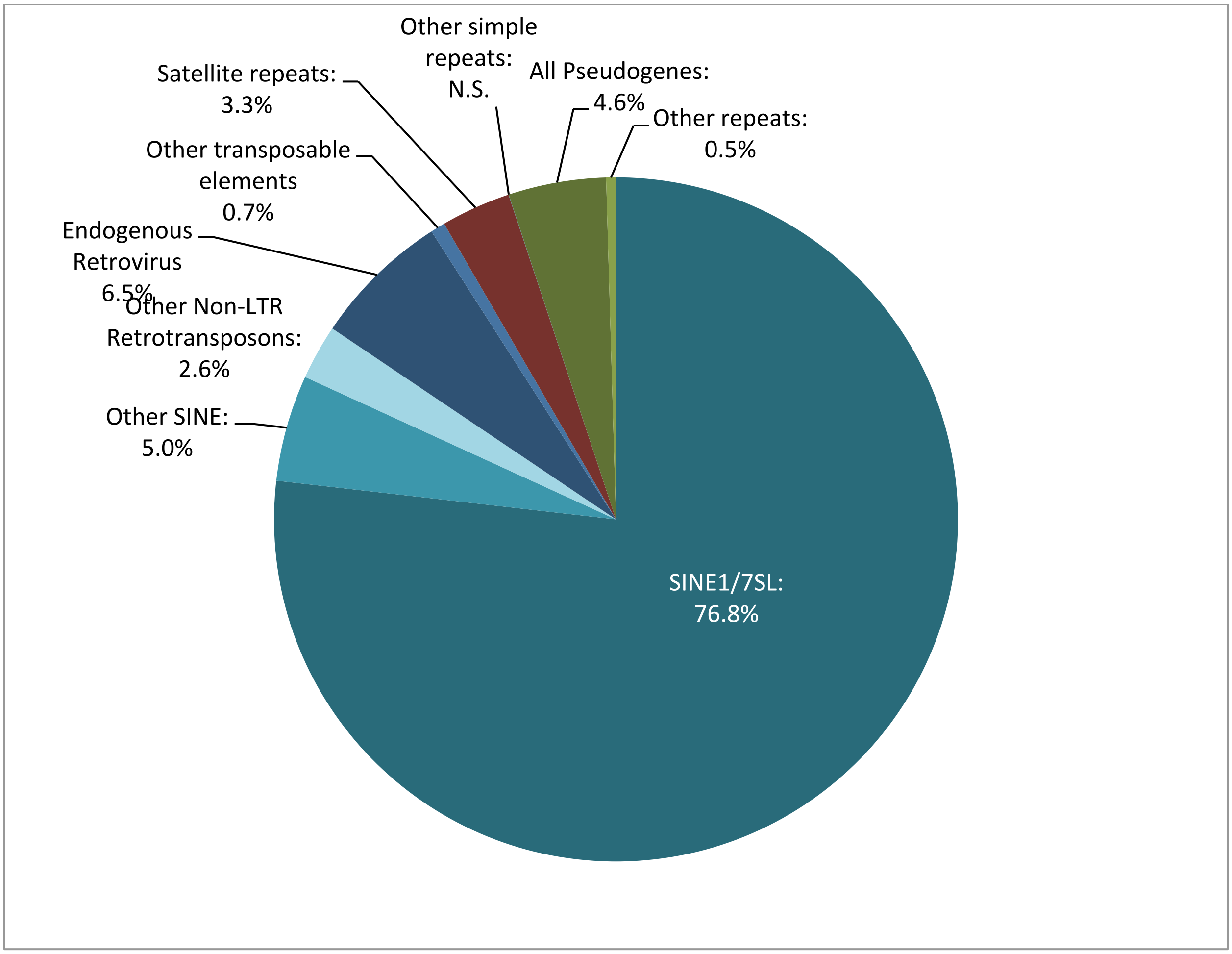

Supplementary Figure 5: Breakdown of repeat elements captured by mRRBS reads. Reads from the twelve mRRBS libraries used to compare coverage of RRBS to mRRBS (Table 2 and Figure 3) were aligned to 1170 human and ancestral repeats from Repbase using MAQ (http://maq.sourceforge.net/). Up to two mismatches were allowed per 29bp read, and only alignments at MspI cut sites were considered valid. Approximately 11.6% of mRRBS reads align to Repbase repeat sequences. The pie chart summarizes the mean fraction of reads aligning to individual repeat element groups. The majority (77%) of reads align to SINE/7SL elements. N.S. denotes a value of <0.1%.

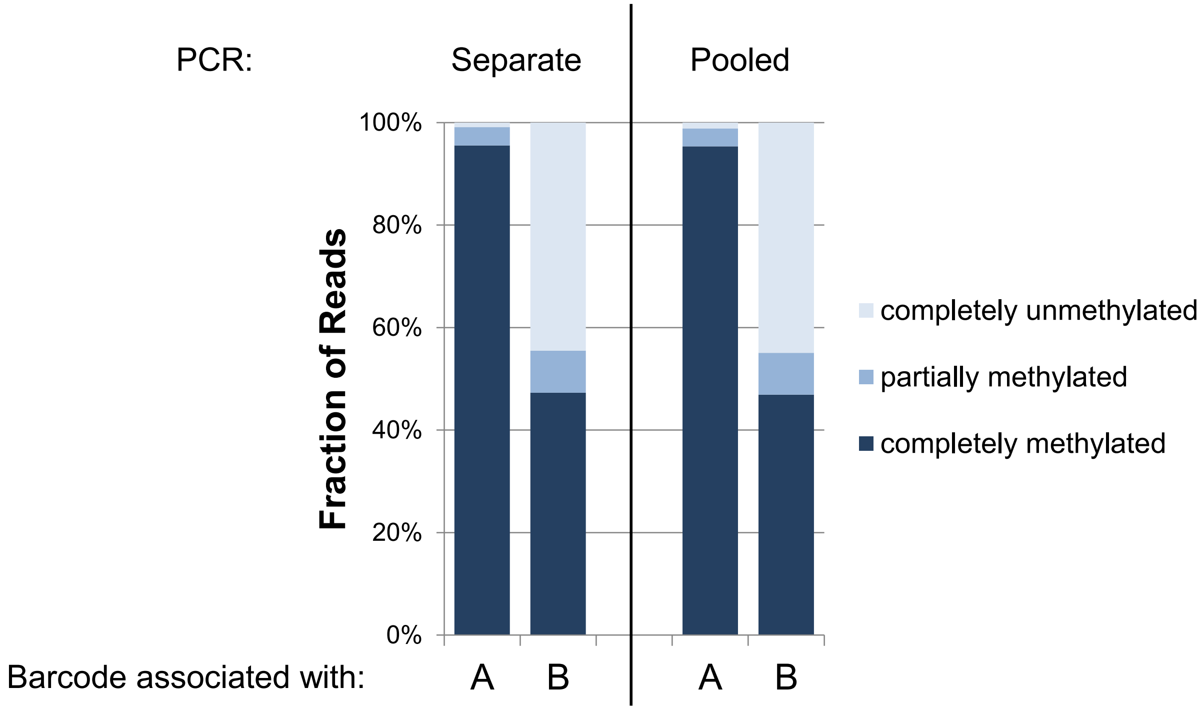

Supplementary Figure 6: Assessment of rate of chimerism during PCR amplification of barcoded RRBS libraries. Barcoded RRBS libraries were constructed from two DNA samples. The PCR amplification was performed either separately (**left**) or as a pool (**right**). Sample A was *in vitro* CpG-methylated mouse DNA. Sample B was mouse DNA from wildtype neuroprogenitor cells (NPC). The proportion of completely methylated (dark blue), completely unmethylated (light blue) and partially methylated reads assigned to each samples based on its respective barcode did not change when the libraries were PCR-amplified as a pool, indicating a negligible rate of PCR-induced chimerism in these mRRBS libraries.
